# The Burden of Mycobacterial Disease in Ethiopian Cattle: Implications for Public Health

**DOI:** 10.1371/journal.pone.0005068

**Published:** 2009-04-07

**Authors:** Stefan Berg, Rebuma Firdessa, Meseret Habtamu, Endalamaw Gadisa, Araya Mengistu, Lawrence Yamuah, Gobena Ameni, Martin Vordermeier, Brian D. Robertson, Noel H. Smith, Howard Engers, Douglas Young, R. Glyn Hewinson, Abraham Aseffa, Stephen V. Gordon

**Affiliations:** 1 TB Research Group, Veterinary Laboratories Agency, Weybridge, New Haw, Addlestone, Surrey, United Kingdom; 2 Armauer Hansen Research Institute, Addis Ababa, Ethiopia; 3 Aklilu Lemma Institute of Pathobiology, Addis Ababa University, Addis Ababa, Ethiopia; 4 Department of Infectious Disease and Microbiology, Imperial College, South Kensington Campus, London, United Kingdom; 5 School of Agriculture, Food Science and Veterinary Medicine, University College Dublin, Dublin, Ireland; 6 School of Medicine and Medical Science, University College Dublin, Dublin, Ireland; 7 School of Biomolecular and Biomedical Science, College of Life Sciences, University College Dublin, Dublin, Ireland; 8 UCD Conway Institute of Biomolecular and Biomedical Research, University College Dublin, Dublin, Ireland; University of Hyderabad, India

## Abstract

**Background:**

Bovine tuberculosis (bTB), caused by *Mycobacterium bovis*, is a debilitating disease of cattle. Ethiopia has one of the largest cattle populations in the world, with an economy highly dependent on its livestock. Furthermore, Ethiopia has one of the highest incidence rates of human extrapulmonary TB in the world, a clinical presentation that is often associated with transmission of *M. bovis* from cattle to humans.

**Methodology/Principal Findings:**

Here we present a comprehensive investigation of the prevalence of bTB in Ethiopia based on cases identified at slaughterhouses. Out of approximately 32,800 inspected cattle, ∼4.7% showed suspect tuberculous lesions. Culture of suspect lesions yielded acid-fast bacilli in ∼11% of cases, with *M. bovis* accounting for 58 of 171 acid-fast cultures, while 53 isolates were non-tuberculous mycobacteria. Strikingly, *M. tuberculosis* was isolated from eight cattle, an unusual finding that suggests human to animal transmission.

**Conclusions/Significance:**

Our analysis has revealed that bTB is widely spread throughout Ethiopia, albeit at a low prevalence, and provides underpinning evidence for public health policy formulation.

## Introduction

Bovine tuberculosis (bTB) is an infectious disease of cattle caused by *Mycobacterium bovis* and characterized by the formation of granulomatous lesions (tubercles) classically seen in the lungs and draining lymph nodes. Infection with *M. bovis* can be transmitted from cattle to humans, mainly through the consumption of contaminated milk and meat products; because of the route of infection, disease often manifests itself as extrapulmonary TB. Indeed, Kidane and colleagues concluded that among 35 PCR-positive cases of tuberculous lymphadenitis from southern Ethiopia, 29 (82.9%) were caused by *M. tuberculosis* and six (17.1%) were caused by *M. bovis* [1]. It is also noteworthy that 36% of incident TB cases in Ethiopia are extrapulmonary [2]. This is exceptionally high, considering that ∼15% of incident TB is extrapulmonary in most high burden countries [2].

Ethiopia has ∼40 million cattle, the largest cattle population in Africa and the seventh in size in the world (Ethiopian Ministry of Agriculture and Rural Development; http://www.moard.gov.et/statstical.htm). Approximately 80% of the labour-force works in agriculture, with this sector accounting for 47% of GDP and with an export value of agricultural goods of about USD $406 million in the year 2000 [3]. The livestock sector is of national importance and the Ethiopian government has set goals to improve productivity in this sector, such as intensification and the importation of “exotic” high performance cattle breeds such as Holstein.

Previous studies on bTB in Ethiopia have shown that prevalence varies depending on husbandry methods, with rural settings showing a lower prevalence compared to intensive dairy farms [4]. However, there have been no studies to date that have addressed the total burden of mycobacterial infection in cattle, nor do we know the molecular types of *M. bovis* that are circulating in Ethiopia. Furthermore, given the large cattle population and endemic bTB problem it is possible that some of the incidence of human extrapulmonary TB is caused by infection with *M. bovis*. While this latter connection has yet to be formally shown, knowledge of mycobacterial strains circulating in Ethiopian cattle is essential baseline data to inform public health policy.

Here we present an extensive study of the prevalence of mycobacterial infection from cattle slaughtered at abattoirs across different eco-epidemiological settings of Ethiopia. Suspect tuberculous lesions were processed for culture, with acid-fast isolates typed using molecular methods to provide definitive strain characterisation. The spatial distribution of strains and molecular types could then be mapped across the eco-epidemiological settings. We show that while infection with *M. bovis* is a significant cause of tuberculous lesions in cattle, other acid-fast bacilli (AFB), notably *M. tuberculosis* and non-tuberculous mycobacteria (NTM), are also considerable causes of infection and disease.

## Materials and Methods

### Study sites

**Figure 1:**
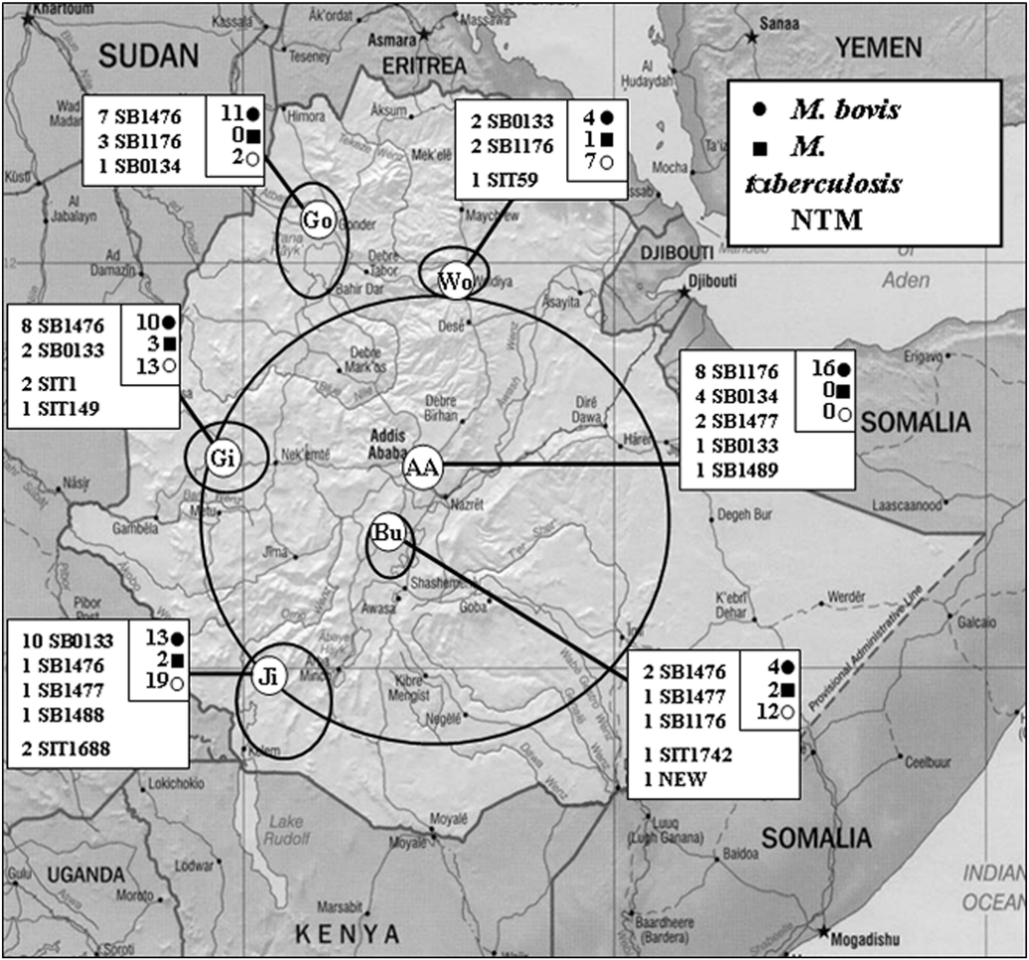
Geographical distribution of *Mycobacterium* isolates from cattle in Ethiopia. The total number of *M. bovis* (•), *M. tuberculosis* (▪), and NTM (○), isolated from respective abattoir are indicated in respective box, as well as characterised spoligotype patterns. Approximate area coverage for each abattoir is shown by a solid circle.

The investigated cattle specimens in this study were collected from abattoirs located in the rural areas of Gonder (abbrev. Go), Woldiya (Wo), Gimbi (Gi), Butajira (Bu), and Jinka (Ji) in Ethiopia (Figure 1). Each geographical area represents a different eco-epidemiological setting, comprising subsistence farming, agro-pastoral, and pastoral communities. People living in agro-pastoral settings are primarily dependent on cattle for farming their land, while pastoral communities are directly dependent on cattle to provide meat and milk products. To complement this study with samples from an urban setting, specimens from lesioned cattle were also collected at the “Enterprise” abattoir in the capital Addis Ababa (AA).

Identifying a precise geographical origin for each investigated animal was not possible. However, there were no nomads in our study areas, suggesting that any cattle movement was due to local, or possibly national, trading. The five rural abattoirs (Gonder, Woldiya, Gimbi, Butajira, and Jinka) documented the markets from where slaughtered animals were purchased by local butchers. Based on this information, and the assumption that no significant cattle movement over large distances was taking place, we determined the approximate area that each abattoir covers (Figure 1). The situation in Addis Ababa is different; the high demand for meat products in the capital necessitates larger supplies. Therefore, merchants buy cattle from a much larger geographical area and transport them to Addis Ababa for slaughter. The area covered by the Addis Ababa abattoir is therefore much greater (Figure 1).

### Sample collection, processing, and culturing

The study obtained ethical clearance from institutional (Armauer Hansen Research Institute (AHRI), All Africa Leprosy, Tuberculosis and Rehabilitation Training Centre (ALERT), Ethiopia; VLA, UK) and Ethiopian national ethical review committees. Trained staff at the six abattoirs performed ante-mortem examination (including sex, breed, and body condition *etc.*) of all or randomly selected cattle that were entering the abattoirs. All ante-mortem examinations were followed up by post-mortem inspections to look for suspect tuberculous lesions in lungs and a range of lymph nodes (hepatic, mesenteric, bronchial, mediastinal, mandibular, and medial retropharyngeal LNs). A description of the lesions (purulent, caseous, or calcified) was recorded. All sampled specimens from respective carcasses were pooled together and kept in phosphate buffer saline (pH 7.2) at 4°C in universal containers and transported on ice to the AHRI laboratory in Addis Ababa within one week. Specimens were processed according to standard methods [5]. In brief, the tissue samples were dissected and manually homogenised using a pestle and mortar, followed by decontamination by shaking in an equal volume of 4% NaOH for 15 min and concentrated by centrifugation at 3,000×g for 15 min. The sediment was neutralized with 2 N HCl, using phenol red as an indicator, and then used to inoculate three different media slants: Löwenstein–Jensen (LJ) media with and without pyruvate, and Middlebrook 7H11 medium supplemented as previously described [6]. The slants were incubated at 37°C for six weeks; slants were examined on a weekly basis for the presence of mycobacterial colonies. Cultures were considered negative if no visible growth was detected after six weeks incubation. Microscopic examination of cultures using the Ziehl–Neelsen staining method was performed to select AFB positive isolates. Heat-killed cells of each isolate were prepared by mixing ∼2 loopfuls of cells (≥20 µl cell pellet) in 200 µl dH_2_O followed by incubation at 80°C for 1 hour. AFB positive cultures were prepared as 20% glycerol stocks and stored at −80°C.

### Molecular typing

Heat-killed AFB positive samples were investigated by multiplex PCR for the presence or absence of RD4, RD9, and TbD1 [7] using the primers listed in Table 1. Oligonucleotide primers used for deletion typing of RD105, RD142, RD150, and RD181 have been described previously [8]. Characterisation of NTM was performed by genus typing [9] and isolates positive for the *Mycobacterium* genus were sequenced at the 16S rDNA locus [10]. 16S rDNA sequences were used in BLAST searches [11] of databases at NCBI and RIDOM (http://rdna.ridom.de)[12]; particularly, the sequences of the hypervariable Region A and Region B [13] were considered when determining the *Mycobacterium* species. The PCR amplification mixtures used for RD and genus typing were as follows: reactions were performed in a total volume of 20 µl consisting of 10 µl HotStarTaq Master Mix (Qiagen, United Kingdom), 7.1 µl distilled H_2_O, 0.3 µl of each oligonucleotide primer (100 µM), and 2 µl DNA template (heat-killed cells, see above). DNA sequencing was performed by The Sequencing Service (College of Life Sciences, University of Dundee, Scotland) using Applied Biosystems Big-Dye Ver 3.1 chemistry on an Applied Biosystems model 3730 automated capillary DNA sequencer. Isolates genetically typed as belonging to the *M. tuberculosis* complex were spoligotyped as previously described [14].

**Table 1:** Oligonucleotide primers used for molecular typing of *Mycobacterium* isolates and sizes of the expected PCR products.

Locus	Primer name	Primer sequence	Present	Deleted
**RD4**	RD4_FlankF	CTCGTCGAAGGCCACTAAAG	335	446
	RD4_FlankR	AAGGCGAACAGATTCAGCAT		
	RD4_InternalF	ACACGCTGGCGAAGTATAGC		
**RD9**	RD9_FlankF	AACACGGTCACGTTGTCGTG	396	575
	RD9_FlankR	CAAACCAGCAGCTGTCGTTG		
	RD9_InternalR	TTGCTTCCCCGGTTCGTCTG		
**TbD1**	TbD1_FlankF	CTACCTCATCTTCCGGTCCA	909	486
	TbD1_FlankR	CATAGATCCCGGACATGGTG		
	TbD1_InternalR	AATCGAACTCGTGGAACACC		
**16S rDNA**	MYCGEN-F	AGAGTTTGATCCTGGCTCAG	1030	NA
	MYCGEN-R	TGCACACAGGCCACAAGGGA		
**16S rDNA** #	16S_AFB_F	GCGTGCTTAACACATGCAAGTC	660	NA
	16S_AFB_R	TCCTCCTGATATCTGCGCATTC		

# Primer set used for partial sequencing of the 16S rRNA locus [10].

NA, Not Applicable.

## Results

### Collection of lesions

In total, about 32,800 cattle were investigated during the years 2006–2008 and the vast majority were of local *Bos indicus* breeds (*i.e.* Zebu). However, *Bos taurus* (*i.e.* Holstein-Frisian) and crossbreeds also occurred among the inspected cattle. At the Gonder, Woldiya, Gimbi and Butajira abattoirs, all of the slaughtered animals were also ante- and post-mortem examined during the period of 2006–2008, while in Jinka approximately 50% of the slaughtered animals were examined. From the end of 2007 to May 2008 the Addis Ababa abattoir investigated approximately 2,800 animals, corresponding to less than 2% of all cattle that passed through the abattoir during this period.

### Culturing and ZN staining results

Approximately 1,500 samples from suspect TB lesions were processed and sown onto the three solid media: 7H11, LJ glycerol, and LJ pyruvate, as described in the Materials and Methods. From the inoculated slants, colonies could be collected at a higher frequency from 7H11 than LJ glycerol or LJ pyruvate (data not shown). In total, 171 AFB were cultured from 1524 lesioned samples (Table 2), with one animal from Gimbi showing dual infection of *M. tuberculosis* and *M. gordonae*.

**Table 2:** Summary of collected lesions and molecular typed AFB isolated from cattle slaughtered at six Ethiopian abattoirs.

Study Site	Animals Investigated	Lesioned Animals	Culture and ZN positive	*M. bovis*	*M. tuberculosis*	NTM*	Total typed
**Butajira**	4606	281	26	4	2	12	18
**Jinka**	3471	278	54	13	2	19	34
**Gimbi**	3250	413	36	10	3	13	26
**Gonder**	14314	261	20	11	0	2	13
**Woldiya**	4338	240	19	4	1	7	12
**Addis Ababa**	2800	51	16	16	0	0	16
**All abattoirs**	32779	1524	171	58	8	53	119

* NTM = Typed as *Mycobacterium* species not from the *M. tuberculosis* complex.

### Characterisation of *Mycobacterium* isolates by molecular typing

Of the 171 AFB isolated, 135 were subjected to a range of molecular typing techniques to determine their identity. To investigate if these isolates were *M. bovis*, deletion typing was performed to verify the specific deletion of the RD4 region in *M. bovis* strains (see Materials and Methods; Table 1). Fifty-eight samples generated a PCR product of 446 bp and were consequently identified as *M. bovis* (Table 2). In the cases where the RD4 region was shown to be intact, RD9 typing was performed to further identify the species. Eight isolates were shown to have both RD4 and RD9 present, suggesting that they were *M. tuberculosis*. Of the remaining AFB positive strains, 53 were typed as NTM, and 16 were not from the *Mycobacterium* genus. The frequency of NTM isolation varied between the collection sites (Table 2). Table 3 shows the NTM strains that were identified by DNA sequencing of the 16S rDNA gene as described in Materials and Methods. Overall, eleven different species were characterised of which *M. nonchromogenicum*, *M. gordonae*, *M. fortuitum*, and *M. peregrinum* were isolated from more than one abattoir.

**Table 3:** Non-tuberculous mycobacteria isolated from cattle in Ethiopia.

Species	Woldiya	Gimbi	Butajira	Jinka
*M. acapulcensis*		1		
*M. colombiense*			1	
*M. engbaekii*		1		
*M. fortuitum*	2			1
*M. gordonae*		3		2
*M. intracellulare*			2	
*M. monacense*		1		
*M. mucogenicum*				1
*M. nonchromogenicum*	1		7	
*M. peregrinum*		1	1	
*M. vaccae*			1	

### Strains of the *M. tuberculosis* complex and their spoligotypes

Strains that were identified as *M. bovis* and *M. tuberculosis* from the six study sites were characterised by spoligotyping and their patterns are listed in Table 4. Strains of *M. bovis* were represented by seven different spoligotype patterns, of which four had not been registered before in the international *M. bovis* spoligotype database at www.Mbovis.org. Of the seven patterns SB0133, SB1476, and SB1176 were more prevalent. Interestingly, tendencies toward geographical clustering could be observed for these spoligotypes (Figure 1). First, SB0133 predominates in Jinka, but it is also represented in Addis Ababa, Gimbi, and Woldiya. The second, SB1476, is the most common pattern in both Gimbi and Gonder but it was found in Jinka and Butajira as well. SB1176 is most prevalent among the samples from Addis Ababa but can also be seen in Butajira, Gonder and Woldiya. The remaining four spoligotype patterns, SB0134, SB1488, SB1489, and SB1477, are all highly related to SB0133, and isolates with these patterns were mostly collected from the Addis Ababa or Jinka abattoirs. The largest diversity of *M. bovis* strains was found in Addis Ababa abattoir with five different spoligotypes, likely reflecting the wide geographical area from which cattle were sourced.

**Table 4:** Spoligotyping patterns of A) *M. bovis* strains and B) *M. tuberculosis* strains isolated from cattle in Ethiopia.

▪	Spoligotype*	Spacers																																											Isolates per site						
		1	2	3	4	5	6	7	8	9	10	11	12	13	14	15	16	17	18	19	20	21	22	23	24	25	26	27	28	29	30	31	32	33	34	35	36	37	38	39	40	41	42	43	AA	Bu	Gi	Go	Ji	Wo	Total
**A)**	SB0134	▪	▪	▪	▪	▪	▪	▪	▪	▪	▪	▪	▪	▪	▪	▪	▪	▪	▪	▪	▪	▪	▪	▪	▪	▪	▪	▪	▪	▪	▪	▪	▪	▪	▪	▪	▪	▪	▪	▪	▪	▪	▪	▪	4			1			5
	SB0133	▪	▪						▪		▪	▪	▪	▪	▪	▪		▪	▪	▪	▪	▪	▪	▪	▪	▪	▪	▪	▪	▪	▪	▪	▪	▪	▪	▪	▪	▪	▪						1		2		10	2	15
	SB1488	▪	▪						▪		▪	▪	▪	▪	▪	▪		▪	▪	▪	▪	▪	▪	▪	▪	▪	▪		▪	▪	▪	▪	▪	▪	▪	▪	▪	▪	▪										1		1
	SB1489	▪	▪						▪		▪	▪	▪	▪	▪	▪		▪	▪	▪	▪	▪	▪	▪	▪	▪	▪	▪	▪	▪	▪	▪		▪		▪	▪	▪	▪						1						1
	SB1477	▪							▪		▪	▪	▪	▪	▪	▪		▪	▪	▪	▪	▪	▪	▪	▪	▪	▪	▪	▪	▪	▪	▪		▪	▪	▪	▪	▪	▪						2	1			1		4
	SB1476	▪	▪		▪	▪	▪	▪	▪		▪	▪	▪	▪	▪	▪		▪	▪	▪	▪	▪	▪	▪	▪	▪	▪	▪	▪	▪	▪	▪	▪						▪							2	8	7	1		18
	SB1176	▪	▪	▪	▪	▪	▪	▪	▪	▪	▪	▪	▪	▪	▪	▪	▪	▪	▪	▪	▪	▪	▪	▪	▪	▪	▪	▪	▪	▪	▪	▪	▪	▪	▪	▪	▪	▪	▪	▪	▪	▪	▪	▪	8	1		3		2	14
B)	SIT1688	▪	▪	▪	▪	▪	▪	▪	▪	▪	▪	▪	▪	▪	▪	▪	▪	▪	▪	▪							▪	▪	▪	▪	▪	▪	▪					▪	▪	▪	▪	▪	▪	▪					2		2
	NEW	▪	▪	▪	▪	▪	▪	▪	▪	▪	▪	▪	▪	▪	▪	▪	▪	▪	▪		▪	▪	▪	▪	▪	▪	▪	▪	▪				▪					▪	▪	▪	▪	▪	▪	▪		1					1
	SIT1742	▪	▪	▪	▪	▪	▪	▪		▪	▪	▪	▪	▪	▪	▪	▪	▪	▪	▪	▪	▪	▪	▪	▪	▪							▪					▪	▪	▪	▪	▪	▪	▪		1					1
	SIT59	▪	▪	▪	▪	▪	▪	▪	▪	▪	▪	▪	▪	▪	▪	▪	▪	▪	▪	▪	▪					▪	▪					▪	▪					▪	▪	▪	▪	▪	▪	▪						1	1
	SIT149	▪	▪	▪	▪	▪	▪	▪	▪	▪											▪	▪	▪	▪	▪	▪	▪	▪	▪	▪	▪	▪	▪					▪	▪	▪	▪	▪	▪	▪			1				1
	SIT1 (Beijing)	▪	▪	▪	▪	▪	▪	▪	▪	▪	▪	▪	▪	▪	▪	▪	▪	▪	▪	▪	▪	▪	▪	▪	▪	▪	▪	▪	▪	▪	▪	▪	▪	▪	▪	▪	▪	▪	▪	▪	▪	▪	▪	▪			2				2

* SB No = www.mbovis.org; SIT No = Spoligo-International-Typing.

Eight *M. tuberculosis* strains were isolated from cattle, and they originated from four out of the six abattoirs. The TbD1 region [7] was deleted in all of these eight isolates. The spoligotype patterns of seven of these isolates had been registered previously in the Spoligo-International-Typing (SIT) database [15] and the majority were of the Euro-American lineage (Table 4). Two of the strains were of the Beijing lineage, based on the characteristic spoligotype pattern (SIT1) and by identification of the lineage specific deletions RD105 and RD181 [16]. However, the RD142 and RD150 regions that are frequently deleted in Beijing strains were present in these two cattle isolates.

## Discussion

The aim of this study was to obtain data on the prevalence and epidemiology of bovine TB in cattle in Ethiopia so as to understand the disease burden and to inform public health policies. Ethiopia has the largest cattle population in Africa, and practices such as the consumption of raw milk and meat mean that the opportunity for zoonotic transmission of bTB is high. However, accurate baseline data for the prevalence of bTB in Ethiopia, across a rage of eco-epidemiological settings, is needed to ensure that public health policies and disease control strategies are based on sound scientific data. We therefore carried out slaughterhouse surveys across major geographical areas of Ethiopia to determine the burden of mycobacterial infection, the prevalence of bTB, and the molecular types of *M. bovis* circulating in Ethiopia.

### The agricultural settings

The societies in the highlands around the abattoirs of Gonder, Woldiya, Gimbi, and Butajira, are supported by farming in agro-pastoral settings. This is in contrast to the lowlands in the southern regions of Ethiopia, to the south of Jinka, where farming in pastoral settings is practised. In rural areas the local Zebu cattle are the dominant breeds, while exotic breeds are rare. However, closer to Addis Ababa, in the centre of Ethiopia, farming is more diverse and exotic breeds - such as Holstein-Frisian - are common in dairy farms. The cattle in dairy farms are usually subject to intensive farming methods as compared to husbandry practices among pastoralists who keep their animals grazing outdoors.

### Lesion prevalence

Results were based on post-mortem examinations of about 32,800 cattle (Table 2), making this one of the largest bovine TB studies of its kind in Africa. About 1,500 of these animals (∼4.7%) carried suspect TB lesions. In 1975 the prevalence of tuberculous lesions at meat inspections in slaughterhouses varied between 0.02–1.83% in different parts of Ethiopia [17], while data from reports in 2003 and 2004 showed an increase to 1.48–5.16% [18], [19]. No culturing of AFB was reported in these latter studies.

### AFB positives

The culture yield of AFB from lesions varied depending on the abattoir they were collected from, but the overall yield from visible lesions was calculated to be ∼11%. This is a relatively low number when compared to other countries such as Great Britain, where typical TB lesions collected at slaughterhouses yield AFB in ∼50% of cases [20]. This may reflect subjective differences in identifying tuberculous lesions across the study sites. From the 135 typed AFB, 58 were *M. bovis*, 8 *M. tuberculosis*, 53 NTM, and 16 could not be identified (Table 2).

### Speciation of NTM

About one third of the isolated AFB positives that were typed using molecular methods in this study were confirmed as NTM. As a comparison, isolation of NTM from cattle at slaughterhouses in Great Britain runs at less than 1%, with the majority of isolates from the *M. avium* complex [21]. This may reflect a different population of NTM in Ethiopia as compared to Great Britain, and differences in husbandry practices.

The 11 species of NTM listed in Table 3 contain both fast and slow growing mycobacteria. Most of these NTM have been collected before from cattle and wildlife animals in different parts of Africa, suggesting that they are widely spread in nature. For example, *M. engbaekii* and *M. vaccae* have been isolated in buffaloes in South Africa [22] and a study from Chad found *M. fortuitum*, *M. nonchromogenicum*, and strain of the *M. avium* complex to be common in cattle and humans [23]. *M. nonchromogenicum* was the most frequently isolated NTM in our study; it has previously been collected from cattle lymph nodes and nasal mucus [24]. Interestingly, *M. nonchromogenicum* has been linked to bacteremia in AIDS patients [25], suggesting a potential risk of transmission from cattle to HIV positive humans. Limited information is available for the three strains *M. acapulcensis*, *M. colombiense*, and *M. monacense*, but the latter two have previously been isolated from humans [26]–[28].

### Isolation of *M. tuberculosis* from cattle

Unexpectedly, 8 *M. tuberculosis* strains were collected from cattle from four different abattoirs, and isolated from lesions defined as purulent or calcified (Figure 1 and Table 2). Most of these cattle strains were of the Euro-American lineage but, interestingly, two of the strains collected from the Gimbi abattoir (but nine months apart) were of the *M. tuberculosis* Beijing lineage, and to our knowledge, this is the first description of this type in Ethiopia. *M. tuberculosis* has been sporadically isolated from cattle in other countries [29], [30] or zoo animals [31]. It is tempting to speculate that the isolation of *M. tuberculosis* from cattle is evidence of transmission from humans. The prevalence of *M. tuberculosis* in humans in Ethiopia is 643 cases per 100,000, the 7^th^ highest in the world [2]; as rural dwellers live in close contact with their animals (*i.e.* sharing their house at night-time), many animals are at risk from human-to-cattle transmission. Future studies are needed to find out if isolation of *M. tuberculosis* from cattle is a “spill over” effect from humans, or if these *M. tuberculosis* strains are adapted to cattle. The latter also raises the question of the risk for cattle-to-cattle and cattle-to-human transmission and the impact it may have on public health.

### Differentiation of *M. bovis* strains by spoligotyping

Fifty-eight *M. bovis* strains were spoligotyped and categorised into seven different types (Table 4) with five of these patterns so far apparently unique to Ethiopia. One of these spoligotypes SB1176 was first identified in a government farm in Holeta close to Addis Ababa [32]. Our data from the Addis Ababa abattoir suggests that SB1176 is the most common spoligotype in the central part of Ethiopia. However, it was also identified among strains isolated from the northern part of Ethiopia (Gonder and Woldiya). The cattle slaughtered in Addis Ababa are traded over a large area of Ethiopia, and as such the mycobacterial strains isolated from these cattle should provide a general picture of strain types in Ethiopia. This is supported by the large diversity of spoligotype patterns that was found at Addis Ababa abattoir.

As well as the five spoligotypes that appear to be unique to Ethiopia, we also identified patterns SB0133 and SB0134. The former is most frequent in Jinka in the southern part of the country. An interesting association is that SB0133 has been isolated from cattle in pastoral areas of nearby Uganda [33] and also from several areas of Tanzania [34]. However, SB0133 has so far not been reported outside Africa. It is therefore possible that SB0133 belongs to an *M. bovis* lineage that has spread over a large geographical area of Eastern Africa. SB0134, on the other hand, is a pattern that seems to be widespread in Europe where it has been found in countries such as Great Britain, France, Spain, and Belgium [35]–[37]. It has also been isolated in Africa; a recent publication from Mali suggests that SB0134 is one of the most common patterns in the country [38]. Further chromosomal analysis of strains from Ethiopia and neighbouring countries will help to resolve the population structure of *M. bovis* in this geographical region, and shed light on how *M. bovis* has evolved in this location.

### Conclusion

Our analysis has revealed that bTB is widely spread throughout Ethiopia, although our rates of *M. bovis* culture-positives from suspect lesions indicate a low prevalence. Indeed, more than 40% of all culture-positives were NTM, underlining the importance of confirming the presence of *M. bovis* in suspect lesions by culture and molecular methods.

There are reported differences in the susceptibility of Zebu and Holstein cattle breeds to bTB [4]. Similarly, rates of bTB in Ethiopia have been shown to be directly related to husbandry, with intensive farming practices driving increased bTB rates [32]. It is possible therefore that the low prevalence of bTB in our study is at least in part explained by the fact that the vast majority of cattle investigated were of Zebu extraction and from agro-pastoral and pastoral settings (*i.e.* non-intensive). To gain a more complete picture of the impact of bTB in Ethiopia our current investigations are focussing on intensive farms in periurban areas.

In terms of the impact of bTB on public health, our results suggest that the high rates of extrapulmonary TB seen in humans in Ethiopia are likely due to a combination of factors that include, but are not solely a consequence of, *M. bovis* transmission from cattle to humans. However, isolation of *M. tuberculosis* from significant numbers of animals calls for further investigations to elucidate the importance of this finding to public health.
