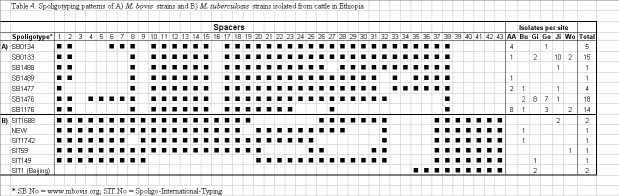# Correction: The Burden of Mycobacterial Disease in Ethiopian Cattle: Implications for Public Health

**DOI:** 10.1371/annotation/f7240b30-f202-45c5-aacb-4fda2efb8b3d

**Published:** 2009-04-28

**Authors:** Stefan Berg, Rebuma Firdessa, Meseret Habtamu, Endalamaw Gadisa, Araya Mengistu, Lawrence Yamuah, Gobena Ameni, Martin Vordermeier, Brian D. Robertson, Noel H. Smith, Howard Engers, Douglas Young, R. Glyn Hewinson, Abraham Aseffa, Stephen V. Gordon

The black square symbols in Table 4 appear incorrectly. Please view the correct Table 4 here:

**Figure:**